# Importance of Seasonal Variation in Hawaiian Mushroom (Agaricomycetes) Basidiomata Production for Biodiversity Discovery and Conservation

**DOI:** 10.3389/ffunb.2022.869689

**Published:** 2022-04-04

**Authors:** Jeffery K. Stallman, Kyra Robinson

**Affiliations:** ^1^Department of Botany and Plant Pathology, Purdue University, West Lafayette, IN, United States; ^2^Department of Biology, University of Hawaii at Hilo, Hilo, HI, United States

**Keywords:** endangered species, fungi, Hawai'i, Hygrophoraceae, Pacific

## Abstract

The Hawaiian Islands have a relatively well-known funga for a tropical location, yet there are over 400 species of mushrooms (Agaricomycetes) in the archipelago that remain to be documented. Importantly, the International Union for Conservation of Nature (IUCN) recently evaluated six mushrooms endemic to the islands as threatened with extinction. To improve detection of mushrooms for biodiversity discovery and better monitor threatened species in the archipelago—where many localities lack strong annual precipitation patterns associated with an obvious season for increased mushroom basidiomata production—we examined the phenology of Hawaiian mushrooms. Monthly richness was determined from a literature review and abundance from online data repositories. Phenological patterns were separately explored for native species and differing elevation and annual precipitation categories. Despite relatively consistent monthly temperatures and areas with regular monthly rainfall, we found Hawaiian mushrooms generally exhibit uneven temporal patterns in basidiomata production: richness and abundance are generally highest in January and lowest from February to April, then usually increase from May to July and remain at elevated levels through December. This pattern does not occur when considering native species richness only, nor when examining abundance data stratified by elevation and annual rainfall categories. Increased monthly basidiomata abundance in low elevation (<1,000 m), dry (<1,000 mm rainfall/year) locations on O‘ahu and low, mesic (1,000–2,500 mm rainfall/year) locations on O‘ahu and Kaua‘i are positively correlated with increased monthly rainfall. Phenology of macrofungal sporocarp production should potentially be included in species threat assessments by the IUCN to increase detection via traditional surveying methods.

## Introduction

Biodiversity discovery of the organisms occurring on earth is ongoing. Lower estimates suggest hundreds of thousands of species still lack formal scientific description (Costello et al., 2012), while others estimate millions of undescribed species within single taxa, such as Arthropoda (Stork, 2018). At the same time, species on earth are facing numerous threats to their existence as many argue earth's biota has entered a sixth mass extinction event, calling for renewed conservation efforts (Ceballos et al., 2015). Fungi, often overlooked in this conversation, have recently been recommended to be included in global conservation goals along with plants and animals (Cao et al., 2021; Gonçalves et al., 2021). They are one of the groups of organisms with the most biodiversity yet to be discovered, with around 135,000 species currently known (Hibbett et al., 2016) out of an estimated 2.2–6.0 million (Taylor et al., 2014; Hawksworth and Lücking, 2017).

Although recently receiving more recognition, the prior exclusion of fungi from these discussions is likely because the majority are microscopic and often undetectable, except through microbial isolation (although many are not culturable with current techniques) or molecular methods. Despite this, some groups such as the class Agaricomycetes (Basidiomycota) are predominantly composed of species that form macroscopic reproductive structures and are considered one of the best-known fungal groups (Kalichman et al., 2020). Most Agaricomycetes basidiomata are recognized as common macrofungi (agarics, boletes, puffballs, corals, polypores, etc.), and therefore are a large proportion of the basidiomata humans interact with for food, recreation, or cultural activities.

Large amounts of data, including life history and phenology information, are required to find mushrooms, evaluate their conservation status, and continue to monitor populations. Having these data provides higher chances of detecting these cryptic organisms, which other than persistent, perennial species, undergo most of their lifecycle as microscopic hyphae immersed in a substrate. For groups such as the Agaricomycetes that form macroscopic reproductive structures, timing of surveys is vital to increase detection (Halme and Kotiaho, 2012).

**Figure 1:**
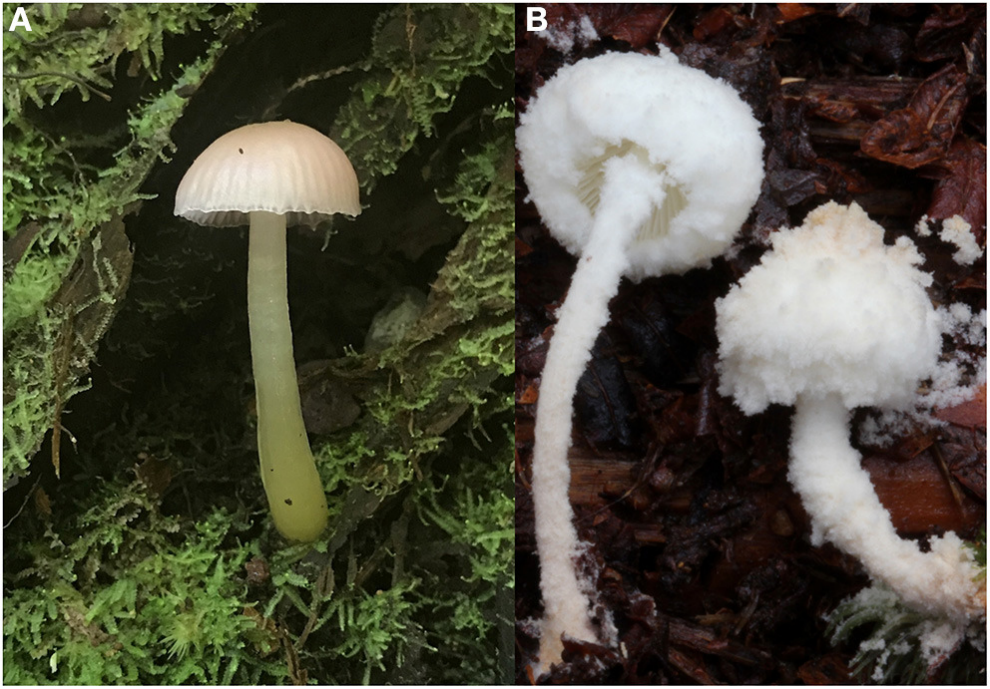
*Hygrocybe noelokelani*, an endemic, endangered Hawaiian mushroom species. Photograph by Amy Durham **(A)**. Endemic, undescribed *Cystolepiota* sp. sensu Hemmes and Desjardin (2002) **(B)**.

In 2017 and 2019, the International Union for Conservation of Nature (IUCN) evaluated six endemic Hawaiian Agaricomycetes species and found that four were vulnerable and two endangered (IUCN, 2021). Phenological data could help with monitoring known populations of threatened species over time, such as the endangered *Hygrocybe noelokelani* (Figure 1A), and assist in planning surveys for finding new populations of these species. Additionally, although the Hawaiian Islands likely have one of the best-known tropical funga among Agaricomycetes, with over 600 species known, an estimated 450 species in this group remain to be documented from the islands (Mueller et al., 2007). Formal descriptions and naming of documented species, such as a *Cystolepiota* sp. from Hemmes and Desjardin (2002) (Figure 1B), are needed in addition to exploration for unknown species lacking any documentation. Therefore, phenological data may help with continued biodiversity discovery in the Hawaiian Islands, where an estimated 80% of native Agaricomycotina species are endemic (Mueller et al., 2007).

While patterns exist in other regions explaining when “mushroom season”—i.e., when basidiomata are most diverse and abundant—occurs, this is often anecdotal and based on cultural practices or correlated with increased seasonal rainfall or temperature (Arnolds and Jansen, 1992; Chacón and Guzmán, 1995). In the Hawaiian Islands, seasonal rainfall variation is low in many areas, particularly wet (>2,500 mm/year) locations, and there is no clear monsoon/dry season dichotomy as in other tropical locations (Giambelluca et al., 2013). Temperatures are above freezing year round except at very high elevations, and Hawaiian cultural practices related to fungi, such as a season for foraging mushrooms, have not been recorded (Hemmes and Desjardin, 2002).

The phenology of Hawaiian Agaricomycetes has had limited study. Hemmes and Desjardin (2002) stated that the best time to find mushrooms is from July through January. Based on a 3-year study of the genera *Pholiota* (1 species), *Hygrocybe* (7 species), and *Rhodocollybia* (1 species) occurring in montane forest environments, these authors found increased abundance of basidiomata from July to December. Stallman (2019) found the highest species richness was from May to January among 38 primarily non-native mushrooms in the family Agaricaceae. Non-native mushrooms are common in disturbed habitats in the Hawaiian Islands and usually associate with non-native plant communities.

To help determine when surveys should occur by conservationists, taxonomists, or community scientists for monitoring known species and continued biodiversity discovery, we investigated the best months of the year for increased basidiomata richness and abundance by performing a literature review and aggregating observation and collection data available in online databases. We examined seasonality based on average monthly temperature and rainfall separately for native (indigenous) species only, and under different elevation (lowland, montane, subalpine/alpine) and annual rainfall (dry, mesic, wet) categories.

We hypothesized, following Hemmes and Desjardin (2002) and Stallman (2019), that Hawaiian Agaricomycetes species would show seasonal patterns in richness in abundance: both would increase in May, June, or July, and remain elevated through January before declining in February, irrespective of average monthly rainfall and temperature, native or non-native origin, and annual rainfall and elevation categories.

## Methods

### The Hawaiian Archipelago

The Hawaiian Islands are a tropical, oceanic archipelago consisting of eight current high islands (Hawai‘i, Kaho‘olawe, Kaua‘i, Lāna‘i, Maui, Moloka‘i, Ni‘ihau, and O‘ahu) in the northern Pacific Ocean. The islands are isolated from the closest land mass, North America, by more than 3,500 km, and have high elevation and rainfall heterogeneity between and within islands. For example, on Hawai‘i Island, average rainfall varies from <300 mm/year in Kawaihae to > 7,500 mm/year at the Makahanaloa rain gauge, and average annual temperature varies from 24°C in Kawaihae at sea level to 4°C on Maunakea at > 4,000 m. In this study, the islands of Ni‘ihau and Kaho‘olawe were excluded from all analyses due to lack of environmental data and historically few collections (e.g., one meeting our criteria from Ni‘ihau; zero from Kaho‘olawe for online abundance data). Additional information on the islands included in this study are available in Supplementary Table 1.

### Richness Analysis

For our richness analysis, we first determined all Agaricomycetes species known to occur in the Hawaiian Islands based on authoritative sources. We then tabulated which month(s) basidiomata of each species were reported in, and additional environmental and life history data from these same sources. The species list of Hawaiian Agaricomycetes was tabulated from an unpublished list of Hawaiian Agaricomycotina compiled by Drs. Dennis Desjardin and Don Hemmes from ~1992 to 2007 and used by Mueller et al. (2007) for their analysis of Hawaiian and global macrofungal biodiversity. We checked primary literature, field guides, aggregated checklists, MyCoPortal.org, and GenBank to verify each species on this list occurred in the Hawaiian Islands, and added species documented since 2007, or those missing. To be included, a reference needed to explicitly state the species occurred in the islands; environmental sequencing studies were not considered. Species documented with incontrovertible DNA evidence and metadata were included, even if unpublished (e.g., *Psilocybe cyanescens*). Mycobank (Crous et al., 2004) and IndexFungorum (www.indexfungorum.org) were used to update fungal names and primary literature was consulted in case of disagreement. Names were compiled at the species level without consideration for infraspecific variation (e.g., subspecies or varieties).

For species on the list, months in which basidiomata were found, different islands they were found on, origin as native (indigenous to the Hawaiian Islands; not inferred to be established via human-mediated dispersal) or non-native (inferred to be established via human-mediated dispersal), and elevation and rainfall information were recorded. To determine a species origin as non-native or native we followed statements in the literature when available or inferred this information from its association with solely native vegetation or habitats. We used a conservative approach, not assigning species to native origin in ambiguous situations in which the species occurs in both native and non-native habitats, or mixed vegetation, unless additional evidence existed. Rainfall and elevation information for each island were compiled based on Hawaiian habitat zones by elevation (lowland < 1,000 m, montane 1,000–2,000 m, and subalpine/alpine > 2,000 m) and rainfall (wet > 2,500 mm, mesic 1,000–2,500 mm, and dry < 1,000 mm) similar to Gagné and Cuddihy (1999) using the Rainfall Atlas of Hawai‘i (Giambelluca et al., 2013) and web resources (https://www.freemaptools.com/elevation-finder.htm) based on reported collection locations. We used a liberal approach for environmental variables, allowing values approximately between two elevation or rainfall categories to be included in both as some location information is not specific (e.g., “Honolulu” or “slopes of Mauna Kea”) and could encompass multiple zones. Additionally, the Hawaiian Islands are known for steep environmental gradients where rainfall can quickly change. We note that temperature throughout the year in the islands, like other tropical locations, is relatively stable and the largest temperature swings generally come from daily cycles as opposed to annual cycles (Hartshorn, 2013). As elevation increases, average air temperatures decrease (Giambelluca et al., 2014).

### Abundance Analysis

For our abundance analysis, we first obtained online observation and/or collection records of Hawaiian fungi identified to the class Agaricomycetes (whether to species, or a higher level), then used their associated geospatial information to extract environmental metadata. We searched for collections and/or observations of Agaricomycetes species in the Hawaiian Islands through May 25, 2021 from iNaturalist.org, MushroomObserver.org, and Mycoportal.org. Observation and collection data were cleaned considering the shortcomings of fungal repository data outlined in Hao et al. (2021): observations or collections that were duplicates, lacked geospatial or temporal data, or fell outside the Agaricomycetes were removed. To be included, associated geospatial coordinates needed to fall on land within the Hawaiian Islands. ESRI ArcGIS (version 10.7.1) was used to visualize observations and collections throughout the Hawaiian Islands and bin these into rainfall and elevation categories. Rainfall data was again obtained from the Rainfall Atlas of Hawai‘i, elevation data was obtained from the National Centers for Coastal Ocean Science Observations (NCCOS, 2022), and temperature data from the Climate of Hawai‘i (Giambelluca et al., 2014). Observations and/or collections that matched species identified as native from the list used for our richness analysis were also indicated as native in our abundance analysis.

Because the habitat in the Hawaiian Islands is highly heterogeneous, correlating increased basidiomata production with environmental variables across the entire archipelago, or even a single island, is difficult. To better investigate whether basidiomata production correlated with average monthly elevation or rainfall, we subset our abundance data into categories by elevation, annual rainfall, and island, as in our richness analysis. Maui, Moloka‘i, and Lāna‘i were considered as a single entity, Maui Nui, as is often done in biogeographical studies due to the connectivity of these islands in the recent past (Price and Elliott-Fisk, 2004). Bins with < 200 data points were excluded. This left wet, lowland habitats on Hawai‘i and O‘ahu; mesic, lowland habitats on Hawai‘i, O‘ahu, and Kaua‘i; and dry, lowland habitats on O‘ahu. Point interpolations were performed in ArcGIS using default settings to determine where the highest density of observations and collections occurred. Average monthly rainfall and temperature from the interpolated highest density region were then obtained from Giambelluca et al. (2013, 2014).

### Excluded Species and Analyses

For both our richness and abundance datasets, the following species were excluded from our analyses as detection may occur year-round due to long-lasting, persistent structures: species characterized as polyporoid (e.g., Polyporales; Hymenochaetales spp.), corticioid (e.g., Corticiaceae spp.), gelatinous (e.g., Auriculariales spp.), or species with otherwise persistent basidiomata (e.g., Nidulariaceae).

We used chi-square tests to determine if Agaricomycetes reproductive phenology varied by month throughout the entire Hawaiian archipelago. The null hypothesis was that no significant difference existed between months in richness or abundance counts across all Agaricomycetes species, and native species only. Pearson's correlation coefficient tests were performed between monthly basidiomata counts of richness and abundance in binned environmental categories with ≥ 200 abundance data points on Hawai‘i Island, Kaua‘i Island, and O‘ahu Island, and the average monthly rainfall and temperature corresponding to the densest location of their abundance. The null hypothesis was that no significant correlation existed between months of increase in basidiomata richness or abundance in a binned habitat (e.g., wet and low elevation) on an island and the monthly temperature or rainfall at the densest location of their abundance. Analyses were conducted using R (R Core Team, 2021).

## Results

### Richness and Abundance Species Lists

Based on our literature review, we found 643 known Agaricomycetes occur in the Hawaiian Islands (Supplementary Table 2). Of the 643 species, 321 had associated seasonality data and lacked long-lasting or persistent reproductive structures to be included in our analysis. Of these, 33 species were considered native in our study and included in the native-only richness analyses.

We downloaded 11,292 observations or collections from iNaturalist.org, MushroomObserver.org, and Mycoportal.org through May 25, 2021. After cleaning, we found 3,285 unique observations or collections meeting our study criteria (Supplementary Table 3). The 3,285 observations or collections contain 478 unique names, although due to the high number of records identified above the species level, the number of unique species these records constitute is unknown. Of these, 169 observations and/or collections of 22 unique species were considered native and included in the native-only abundance analyses.

### Seasonality Trends and Analyses

**Figure 2:**
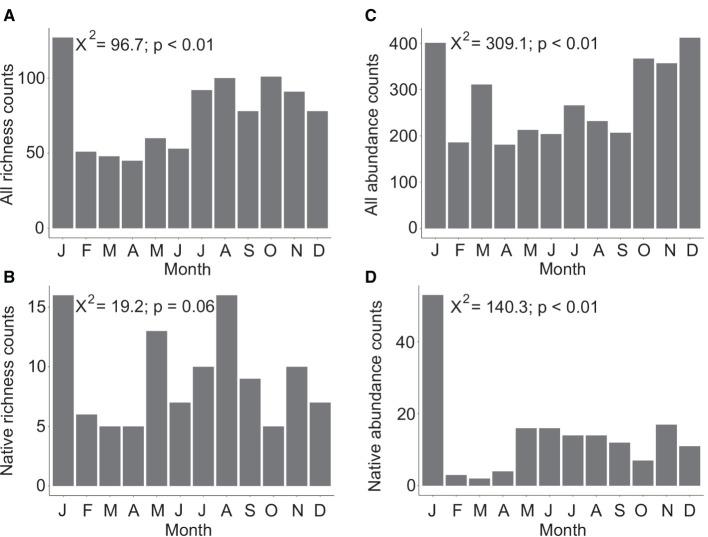
Total species diversity (richness) across 12 months considering all Agaricomycetes included in this study (*n* = 321) **(A)**, and only native species (*n* = 33) **(B)**. Total observations and collections aggregated from online resources (abundance) across 12 months considering all Agaricomycetes (*n* = 3,285) **(C)**, and only native species (*n* = 169) **(D)**. In both datasets, species known to produce long-lasting, persistent basidiomata were excluded. Chi-square values and *p*-values are indicated in each plot with the null hypothesis being that there is no difference in richness or abundance counts between months.

Overall Agaricomycetes richness shows a peak in January with lower diversity from February to June. Richness increases in July and continues at an elevated rate through December (Figure 2A). A significant difference in richness between months was found (*X*^2^ = 96.7, *p* < 0.01, *df* = 11). Native-only richness show that February to April is a period of lower mushroom diversity with June and October also having low diversity (Figure 2B). For richness counts of native species only, the null hypothesis that there is no difference in richness between the 12 months cannot be rejected (X-squared = 19.2, *p* = 0.06, *df* = 11).

Overall abundance data shows peaks in January, March, and October to December (Figure 2C). A significant difference in species abundance between months was found (*X*^2^ = 309.1, *p* < 0.01, *df* = 11). Native-only abundance data show that January has very high abundance, February to April have low abundance, and May to December have a low baseline of consistent abundance counts (Figure 2D). A significant difference in species abundance between months was found considering native species only (*X*^2^ = 140.3, *p* < 0.01, *df* = 11).

Richness data by rainfall and elevation categories generally show a trend of high diversity in January, low diversity from February to April, May, or June, then high diversity for the remainder of the year with outliers being dry, and subalpine/alpine locations that also having high diversity in March (Supplementary Figure 1). Abundance data by rainfall amount and elevation do not follow an obvious pattern, although abundance is generally highest from October through March (Supplementary Figure 2).

**Figure 3:**
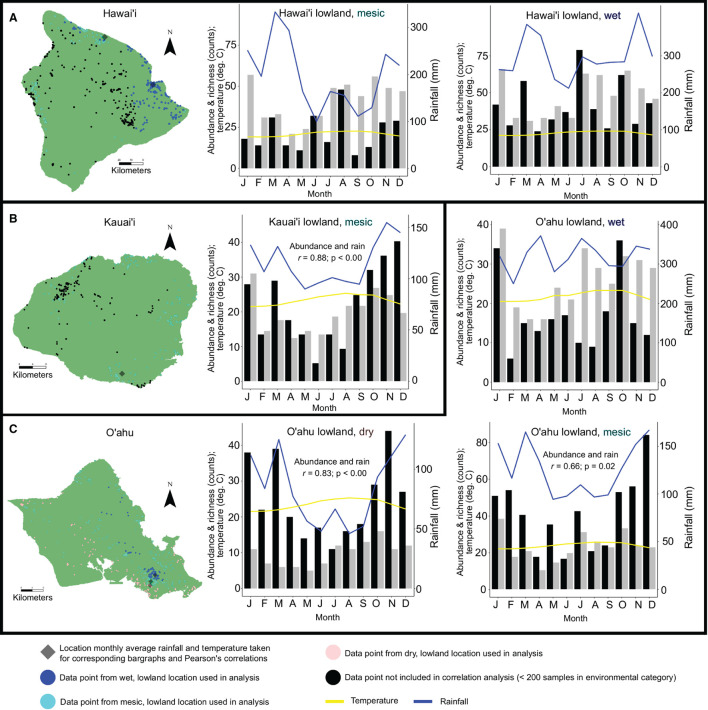
Examination of abundance data with ≥ 200 observations and/or collections and richness data on an island within annual rainfall and elevation categories by average monthly rainfall and temperature. Datapoints are color coded by annual rainfall and elevation categories. The closest climate station to the highest density of data points for a given category that was used to determine average monthly rainfall and temperature is indicated. Histograms show abundance (black), richness (gray), average monthly rainfall (blue) and temperature (yellow) for: Hawai‘i Island, lowland wet and lowland mesic habitats **(A)**; Kaua‘i Island, lowland mesic habitats **(B)**; O‘ahu Island, lowland wet, mesic, and dry habitats **(C)**. Pearson's correlation coefficients between richness and abundance counts and average monthly rainfall or temperature that were found to be significant (*p* < 0.05) are indicated in histogram panels.

Pearson's correlation coefficient tests show significant positive correlations between basidiomata abundance and average monthly rainfall in lowland, mesic environments on Kaua‘i (*r* = 0.88; *p*-value < 0.01) and O‘ahu (*r* = 0.66; *p* = 0.02), and lowland, dry environments on O‘ahu (*r* = 0.83; *p*-value < 0.01). Correlations between abundance of basidiomata and average monthly rainfall or temperature in lowland, wet environments on Hawai‘i Island and O‘ahu, and lowland, mesic environments on Hawai‘i Island were not significant. Correlations between species richness and average monthly rainfall or temperature in either elevation or annual rainfall category were not significant (Figure 3; Supplementary Table 4).

## Discussion

### Trends in Seasonality and Environmental Correlates

Despite a warm climate and areas with consistent year-round rainfall, we found Hawaiian mushrooms generally exhibit seasonality patterns in richness and abundance, except in native-only species richness where we could not reject our null hypothesis (Figure 2). Our results incorporating greater taxonomic breadth agree with the prior findings of Stallman (2019) for richness in the family Agaricaceae, which is primarily represented by non-native species in the islands. Comparing our results of native abundance data to that of Hemmes and Desjardin (2002), we found the month of January to be by far the most abundant, which was one of their least abundant. Additionally, we found native-only basidiomata production is as abundant from May–June as July–December, but May and June showed low abundance in their dataset. These differences could be due to overall low detection of native species, uneven collecting efforts in different locations, or species selection. For example, Hemmes and Desjardin (2002) found *Rhodocollybia laulaha* to be the most abundant native species in their study, but its origin is now uncertain after it was found in the American tropics Keirle et al. (2010). Its origin was assigned as unknown in this study, and therefore not included in our native-only analysis.

Our hypothesis that abundance and richness of mushroom species would not correlate to monthly average rainfall or temperature is supported when considering species richness and elevation, but not for all instances when considering abundance and rainfall. In lowland, dry and mesic environments on O‘ahu, and lowland mesic environments on Kaua‘i, basidiomata abundance is correlated to increased rainfall. Rainfall as a limiting factor to increased abundance in areas lacking consistently high year-round precipitation is intuitive, and follows anecdotal observations and prior studies (e.g., Chacón and Guzmán, 1995). In wet areas with the highest concentration of observations and collections on Hawai‘i and O‘ahu, monthly rainfall is a minimum of 200–300 mm, and increasing rainfall above this baseline does not lead to increased monthly abundance or richness (Figures 3A,C). Increased rainfall in lowland mesic areas on Hawai‘i Island is not positively correlated with increased abundance in basidiomata production despite O‘ahu and Kaua‘i seeing increased production during wetter months in mesic locations (and O‘ahu in lowland dry areas). The reason for this is unclear, but Hawai‘i is a large island (10,430 Km^2^) and by only examining rainfall at the location interpolated to have the highest number of mesic observations and/or collections, accuracy may be lower than for the smaller islands of Kaua‘i (1,456 Km^2^) and O‘ahu (1,545 Km^2^) (Supplementary Table 1).

### Limitations

Several limitations exist in our study. First, our data incorporates several broad categories. Temporally we examined monthly instead of weekly; elevation by 1,000 m, and rainfall by 1,000–1,500 mm. Additionally, we have not considered how seasonality of Agaricomycetes may differ between substrates (e.g., wood or soil) or ecological roles (e.g., saprotroph or symbiotroph), which have been important explanatory variables in a Japanese oak forest (Sato et al., 2012) and across Europe (Andrew et al., 2018).

Second, the majority of Hawaiian Agaricomycetes lack a DNA barcode sequence to help verify species identity and allow quick biogeographical comparisons with other sequence data in GenBank. Our understanding of which species are native may change as more data become available. Additionally, in our abundance analysis, collections and/or observations not identified to the specific level were deemed non-native, although a small portion of these may constitute native species.

Third, our abundance data aggregated from MyCoPortal.org, iNaturalist.org, and MushroomObserver.org may hold biases such as focus on or near population centers, and increased documentation of conspicuous (large, colorful) over inconspicuous species (Gonçalves et al., 2021). Non-native mushrooms often occur near population centers and may have additional water inputs from irrigation. While we believe that most Hawaiian Agaricomycetes collections in herbaria have been digitized (at SFSU, BISH, and ARIZ), some collections at these locations and other herbaria may not be digitized, representing an untapped data source in our abundance analysis that was not captured via MyCoPortal.org searches.

Finally, our data is biased toward the recent past: only 14% of collections or observations in our abundance analysis occurred before 1990, and 29% were before 2000. As climate change has been shown to change macrofungal reproductive phenology (Andrew et al., 2018), some signal in seasonality patterns may be lost due to changes over time in a changing climate. With our data's strong bias toward the present, we are likely seeing less of this effect.

### Conservation Implications

**Figure 4:**
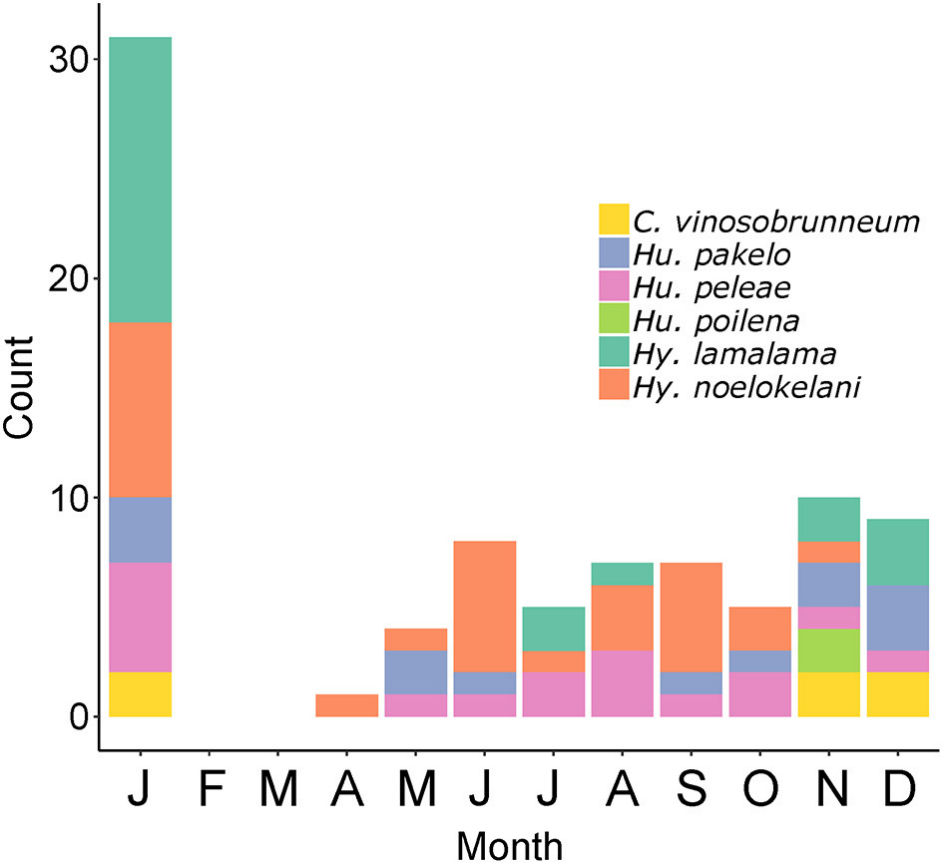
Monthly counts of 87 collections or observations of the six Hawaiian Agaricomycetes species found to be threatened by the IUCN: *Callistosporium vinosobrunneum, Humidicutis pakelo, Humidicutis peleae, Humidicutis poilena, Hygrocybe lamalama, Hygrocybe noelokelani*. Data from MyCoPortal.org, iNaturalist.org, and MushroomObserver.org.

The six Hawaiian fungi that have so far been assessed for the IUCN Red List and were found to be threatened are the endangered *Hygrocybe noelokelani* and *Hy. pakelo* (Vellinga, 2017b, 2019d), and the vulnerable *Callistosporium vinosobrunneum, Humidicutis peleae, Humidicutis poilena*, and *Hy. lamalama* (Vellinga, 2017a, 2019a,b,c). Regarding richness of these species, some show strong seasonality (or rareness in general) with *C. vinosobrunneum* only being found November to January and *Hu. poilena* only in November, while other species, such as *Hu. peleae*, occur throughout much of the year. These threatened species are most abundant in January, November, and December (Figure 4). Although current funds are not allocated to Agaricomycetes conservation by the state or federal government, understanding seasonal basidiomata production could help managers plan field efforts to monitor these species or find additional populations, as is done for imperiled Hawaiian plants (e.g., Kawelo et al., 2012). Although morphological identification of fungi is not always possible (Taylor et al., 2006) or may require experts or DNA data, some threatened species in the Hawaiian Islands such as *Hy. noelokelani* are easily recognizable without molecular identification (Vellinga, 2017b).

Molecular tools can also be used to monitor populations of threatened fungi (Gordon and Norman, 2015). The first barrier to detection of threatened Hawaiian fungal species via these methods is generating reference sequences for the threatened species; currently none have an associated DNA barcode (Schoch et al., 2012). Additionally, substrate would need to be carefully considered as many threatened Hawaiian species form basidiomata on mosses, making it unclear if high throughput sequencing of soil would lead to detection if nearby populations were present.

We note that all threatened Hawaiian Agaricomycetes species are most often found in montane, wet, and mesic habitats which are dominated by the native ‘ōhi‘a tree (*Metrosideros polymorpha*). ‘Ōhi‘a are being killed by the introduced pathogens *Ceratocystis lukuohia* and *C. huliohia*, together known as Rapid ‘Ōhi‘a Death (ROD). The disease has killed over one million trees in the islands (ROD working group, personal communication) and will likely have a detrimental impact on associated native plant communities (Fortini et al., 2019). While direct evidence of native fungal population declines from this disease is lacking, monitoring of threatened fungi is needed to determine demographic and distribution changes as Hawaiian forests change due to the effects of ROD.

## Conclusion

A diverse, abundant number of mushrooms occur throughout the year in the Hawaiian Islands, but richness and abundance are generally highest in January. If general surveys for Agaricomycetes are to be completed and cannot be done year-round, the period from July to January can be recommended for overall elevated richness and abundance, including elevated abundance of native species. Despite this recommendation, richness of native species does not exhibit a strong seasonality pattern as they are found relatively evenly throughout the year, albeit in low abundance.

More selective approaches can be employed for surveys depending on location. In particular, surveys in lowland, dry and mesic environments where increased basidiomata abundance is correlated with increased monthly rainfall (at least on the islands of O‘ahu and Kaua‘i) should be planned accordingly.

The ability of conservationists and community scientists to identify some of these fungi in the field and the temporal variability among species suggests that including seasonality data on macroscopic fungi in IUCN evaluations may be useful to increase detections. This information could be added to the existing ‘Habitat and Ecology' field. Data generated by mycologists and community scientists can be useful in determining the full ranges of threatened species and the phenology of their basidiomata production, particularly in organisms recognizable to the species level by macromorphology. Being able to record the absence of species (i.e., negative records) in online community science platforms would also be useful in gathering range data on threatened species. Collecting baseline seasonality and range data will allow for future comparisons with a changing climate, and generating reference DNA barcodes for threatened species allows for potential monitoring and new population discovery via environmental DNA studies and comparisons with online databases.

## Data Availability Statement

The original contributions presented in the study are included in the article/Supplementary Material, further inquiries can be directed to the corresponding author.

## Author Contributions

JS conceived of the study, completed the research, analyzed the data, and wrote the manuscript. KR completed the research, analyzed the data, and wrote the manuscript. Both authors contributed to the article and approved the submitted version.

## Funding

This article was funded in part by Purdue University Libraries Open Access Publishing Fund.

## Conflict of Interest

The authors declare that the research was conducted in the absence of any commercial or financial relationships that could be construed as a potential conflict of interest.

## Publisher's Note

All claims expressed in this article are solely those of the authors and do not necessarily represent those of their affiliated organizations, or those of the publisher, the editors and the reviewers. Any product that may be evaluated in this article, or claim that may be made by its manufacturer, is not guaranteed or endorsed by the publisher.

## Supplementary Material

Supplementary Table 1 Location, size, maximum elevation, age, and average minimum and maximum annual rainfall and temperature on the six Hawaiian Islands considered in this study.

Supplementary Table 2 List of 643 Agaricomycetes species occurring in the Hawaiian Islands tabulated from sources outlined in methods used in our richness analysis. Origin, association with native or non-native vegetation, annual elevation and temperature categories, island(s) of occurrence, month(s) basidiomata have been documented, and a non-exhaustive list of references are included.

Supplementary Table 3 List of 3,285 observations and collections of Agaricomycetes species from the Hawaiian Islands in online repositories downloaded from MyCoPortal.org, iNaturalist.org, and MushroomObserver.org as of May 25, 2021 (and curated as indicated in methods) used in our abundance analysis. Scientific name, month documented, database downloaded from with unique identifier, location, year of observation, and annual elevation and precipitation categories are included.

Supplementary Table 4 Correlation coefficients between species richness and abundance counts in annual rainfall and elevation categories on an island with average monthly rainfall or temperature at the densest location of those observations and/or collections. Correlations were only performed in annual rainfall and elevation categories on a single island with ≥ 200 observations and/or collections in our abundance dataset.

Supplementary Figure 1 Total Agaricomycetes species diversity (richness) in the Hawaiian Islands over different annual rainfall (wet ≥ 2,500 mm/year, mesic 1,000–2,500 mm/year, dry < 1,000 mm/year) and elevation (lowland < 1,000 m, montane 1,000–2,000 m, subalpine and alpine > 3,000 m) categories, excluding species with persistent reproductive structures (n = 321).

Supplementary Figure 2 Total Agaricomycetes abundance in the Hawaiian Islands over different annual rainfall (wet ≥ 2,500 mm/year, mesic 1,000–2,500 mm/year, dry < 1,000 mm/year) and elevation (lowland < 1,000 m, montane 1,000–2,000 m, subalpine and alpine > 3,000 m) categories based on curated observation and collection data from MyCoPortal.org, iNaturalist.org, and MushroomObserver.org (n = 3,285).
