# Supplementary figures and images for: Importance of Seasonal Variation in Hawaiian Mushroom (Agaricomycetes) Basidiomata Production for Biodiversity Discovery and Conservation
Source: Front Fungal Biol. 2022 Apr 4;3:869689. doi: 10.3389/ffunb.2022.869689 (PMC10512390; doi:10.3389/ffunb.2022.869689)

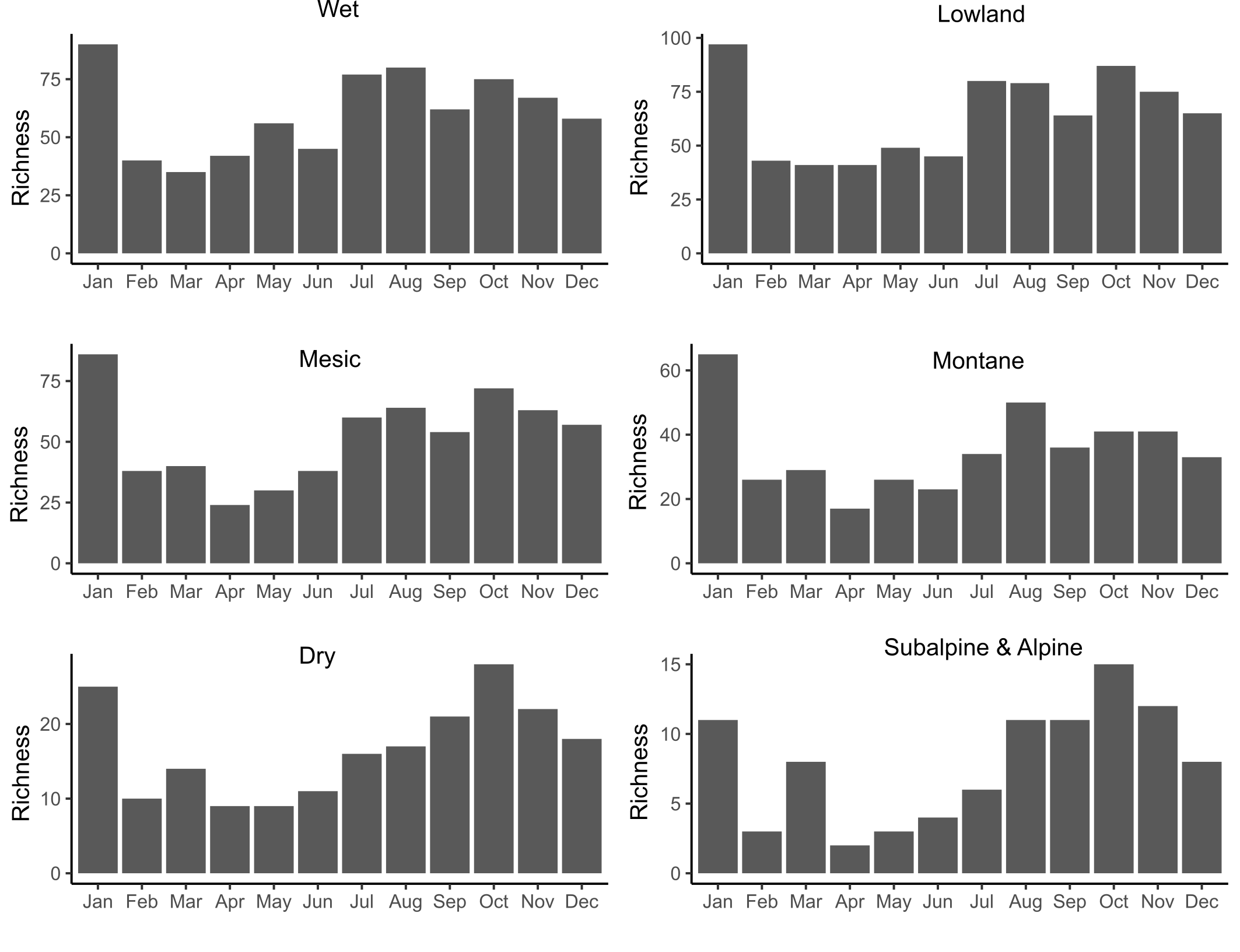

Supplement: Supplementary Figure 1 — Total Agaricomycetes species diversity (richness) in the Hawaiian Islands over different annual rainfall (wet ≥ 2,500 mm/year, mesic 1,000–2,500 mm/year, dry < 1,000 mm/year) and elevation (lowland < 1,000 m, montane 1,000–2,000 m, subalpine and alpine > 3,000 m) categories, excluding species with persistent reproductive structures (n = 321). [file Image_1.TIF]

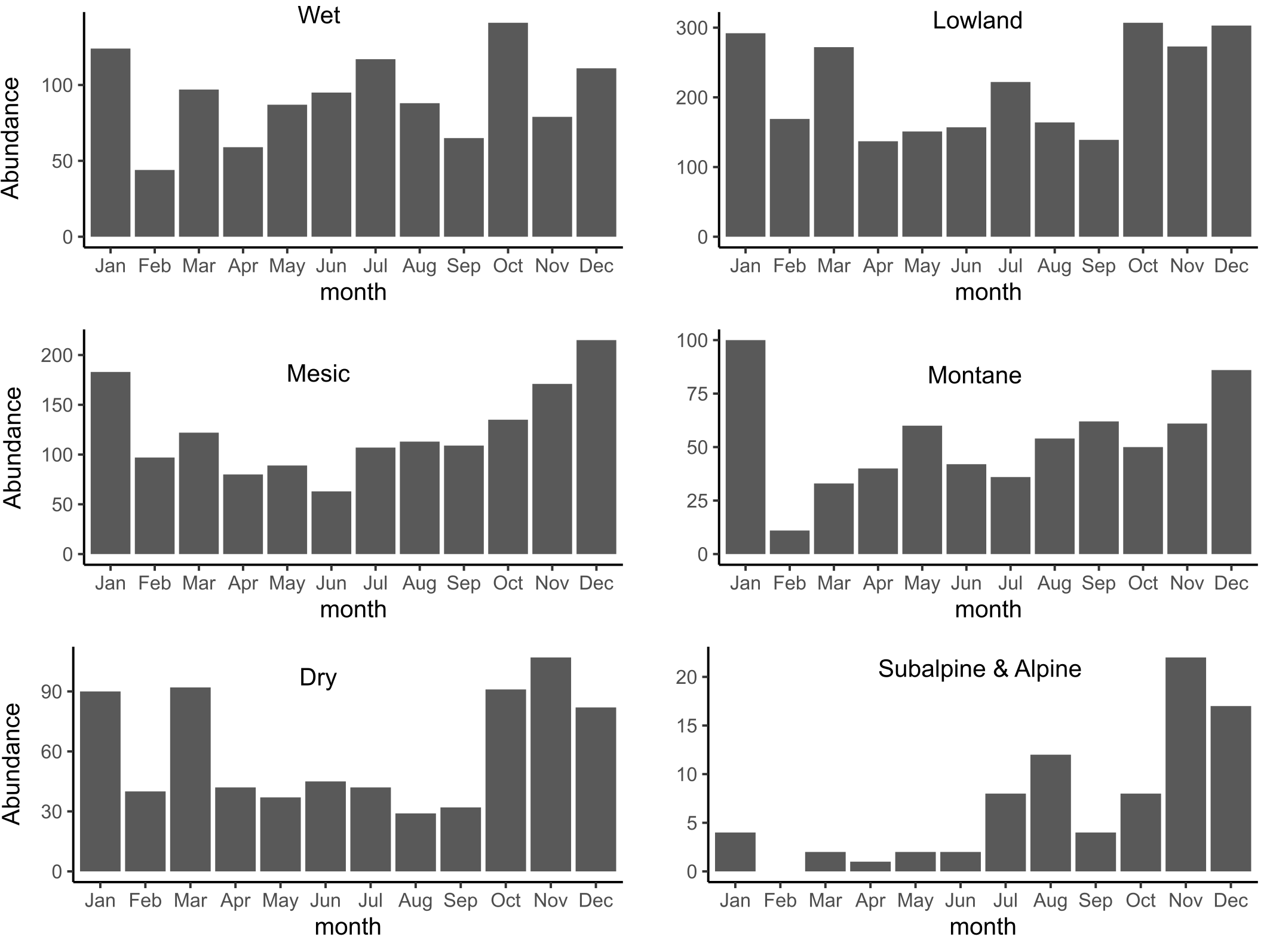

Supplement: Supplementary Figure 2 — Total Agaricomycetes abundance in the Hawaiian Islands over different annual rainfall (wet ≥ 2,500 mm/year, mesic 1,000–2,500 mm/year, dry < 1,000 mm/year) and elevation (lowland < 1,000 m, montane 1,000–2,000 m, subalpine and alpine > 3,000 m) categories based on curated observation and collection data from MyCoPortal.org, iNaturalist.org, and MushroomObserver.org (n = 3,285). [file Image_2.TIF]
